# Role of CD38 in Adipose Tissue: Tuning Coenzyme Availability?

**DOI:** 10.3390/nu13113734

**Published:** 2021-10-23

**Authors:** Andrea Benzi, Alessia Grozio, Sonia Spinelli, Laura Sturla, Andreas H. Guse, Antonio De Flora, Elena Zocchi, Joerg Heeren, Santina Bruzzone

**Affiliations:** 1 DIMES-Section of Biochemistry, University of Genova, 16132 Genova, Italy; andreeabenzi@gmail.com (A.B.); sonia.spinelli94@libero.it (S.S.); laurasturla@unige.it (L.S.); toninodf@unige.it (A.D.F.); ezocchi@unige.it (E.Z.); 2 Buck Institute for Research on Aging, Novato, CA 94945, USA; agrozio@buckinstitute.org; 3 Department of Biochemistry and Molecular Cell Biology, University Medical Center Hamburg-Eppendorf, 20246 Hamburg, Germany; guse@uke.de (A.H.G.); heeren@uke.de (J.H.)

**Keywords:** nicotinamide adenine dinucleotide, CD38, adipose tissue

## Abstract

Nicotinamide adenine dinucleotide (NAD^+^) is a fundamental molecule in the regulation of energy metabolism, representing both a coenzyme and a substrate for different NAD^+^ degrading enzymes. Among these enzymes, CD38 can be seen under two perspectives: as the enzyme synthesizing Ca^2+^-mobilizing second messenger, starting from NAD^+^, and as the major NAD^+^-consumer, to be inhibited to increase NAD^+^ levels. Indeed, the regulation of NAD^+^ availability is a key event during different processes. In this review, we examine the recent studies related to the modulation of CD38 expression and activity, and the consequent changes in NAD(P)(H), in adipose tissue, during inflammation and cold-induced thermogenesis.

## 1. Introduction

Nicotinamide adenine dinucleotide (NAD^+^) is the necessary coenzyme in many redox reactions governing energy metabolism [1]. NAD^+^ also represents a fundamental signaling molecule, regulating different cellular processes, being the substrate of different classes of NAD^+^-consuming enzymes: ADP-ribosyl cyclases/NAD^+^-ase (CD38), sirtuins (SIRT; NAD^+^-dependent deac(et)ylases), poly-ADP-ribose polymerases and mono-ADP ribosyl transferases [2]. This review is focused on the recent evidence of a role for CD38 in adipose tissue, during aging, inflammation, and browning.

### 1.1. CD38 and Signaling Mediated by NAD^+^-Derived Second Messengers

CD38 is expressed in most cell types and it is a multifunctional enzyme, catalyzing: (i) the conversion of NAD^+^ to cyclic adenosine diphosphoribose (cADPR) and adenosine diphosphoribose (ADPR); (ii) the hydrolysis of cADPR to ADPR; (iii) the conversion of 2′-deoxy-NAD^+^ to produce 2′-deoxy-ADPR (2dADPR) [3]; (iv) base exchange reaction converting NAD^+^ to (ADPR)2, by inserting an ADPR moiety [4]; (v) the synthesis of three adenylic dinucleotides (diadenosine diphosphate and two isomers thereof) from cADPR and adenine [5]. In addition, CD38 catalyzes, at acidic pH, a base exchange reaction converting NADP^+^ to NAADP^+^, but it is questionable whether CD38 synthesizes NAADP in intact cells, since it also catalyzes the breakdown of NAADP^+^ to 2-phospho-ADPR [6,7,8]. Anyway, most of the molecules produced by CD38 act as Ca^2+^-mobilizing second messengers, and the role for CD38 as an enzyme regulating different Ca^2+^-mediated signaling pathways has been extensively investigated in different cell types and on several cellular functions [9,10,11]. The cADPR evokes Ca^2+^ release from ryanodine receptors (RyR); ADPR and 2dADPR activate extracellular Ca^2+^ influx through transient receptor potential melastatin 2 (TRPM2), a ligand-gated, Ca^2+^-permeable, nonselective cation channel [10,12].

The inhibition of CD38 enzymatic activity, blocking the CD38-mediated Ca^2+^ signaling, has been proposed as a pharmacologic strategy in different conditions, including, but not limited to, allergic airway disease [13], cardiovascular diseases [14], vascular thrombosis, disordered inflammation, and aberrant immune reactivity [15]. Nevertheless, the best-known field of application of CD38 as a pharmacological target is arguably represented by hematological malignancies: different anti-CD38 monoclonal antibodies have been developed and are currently in use or in clinical trials, especially in multiple myeloma [16,17]. Not all of the antibodies are inhibiting the enzymatic activity of CD38, and they trigger cell cytotoxicity with different mechanisms. In chronic lymphoid leukemia the block of the CD38-mediated signaling cascade has been demonstrated to represent a possible therapeutic strategy [18]. Overall, the role of CD38 in hematological malignancies has been extensively studied, whereas the impact of targeting CD38 within solid tumors is less known. Studies aimed at characterizing the infiltrating immune cell types expressing CD38 and identifying the most promising CD38 inhibitors/antibodies to use, are urgently needed [19]. For the aspects related to the potential CD38 targeting in cancer therapy, outside of the scope of this review, we refer to the recently published review by Morandi et al. [17].

### 1.2. CD38 and Cell NAD^+^ Content

Beside the studies focusing on the role of CD38 in the production of NAD^+^-derived Ca^2+^ mobilizing second messengers, modulation of CD38 activity has been proposed as an effective means to affect NAD^+^ levels. Indeed, NAD^+^ levels decrease during aging, obesity and obesity-related cardiometabolic diseases, in different tissues/organs, including liver and white adipose tissue (WAT) [20,21,22,23]. Thus, several strategies have been investigated as means to boost cellular NAD^+^ levels and thereby to promote healthy aging and extend life spans [24,25]. Increasing intracellular NAD^+^ levels by the systemic administration of NAD^+^ biosynthetic precursors, such as nicotinamide riboside (NR) and nicotinamide mononucleotide (NMN) is one of the most promising approaches that has been extensively investigated in several pre-clinical studies and in human clinical trials [26,27]. Recently, dihydronicotinamide riboside (NRH) was demonstrated to be a natural precursor for the synthesis of NAD^+^, via an NRH salvage pathway involving the enzyme adenosine kinase [28]. In addition, a reduced form of NMN (NMNH) was also identified as a new NAD^+^ precursor, more efficient than NMN or NR at increasing intracellular NAD^+^ concentrations in many organs and tissues: NMNH administration in mice determined the rise of NAD^+^ levels in liver, kidney, muscle, brain, brown adipose tissue, and heart, but not in white adipose tissue [29].

In this review, we examine the recent studies related to the role of CD38 in NAD^+^ metabolism, focusing on the effects of CD38 activity and modulation in adipose tissue, both white and brown; therefore, we do not detail the studies concerning the administration of NAD^+^ precursors and we refer the reader to more comprehensive reviews on this topic [1,26,30].

### 1.3. Adipose Tissue

Adipose tissue (AT) is one of the largest organs, now considered an endocrine organ, and plays an important role in energy balance, and glucose and lipid homeostasis. Two types of AT are present in mammals, white adipose tissue (WAT) and brown adipose tissue (BAT) [31].

Most adipose tissue depots are WAT, with rather large adipocytes containing a single large lipid droplet. Brown adipocytes in BAT have a lower size and contain smaller lipid droplets and more mitochondria. A main function of white adipose tissue (WAT) is the storage of energy, in the form of triglycerides. When energy levels decrease, the stored triglycerides are hydrolyzed and glycerol and fatty acids are released, so that they can be used as energy sources by other organs. WAT is also an important endocrine organ, releasing adipokines, such as leptin and adiponectin that regulate important functions in other tissues, including the brain. The excessive abundance of WAT, as a result of increased energy intake and/or reduced energy expenditure, causes obesity, one of the major public health emergencies in developed societies, often associated with metabolic complications including dyslipidemia, insulin resistance, type 2 diabetes, fatty liver disease, cardiovascular disease, and cancer [32].

Brown adipose tissue (BAT) differs from WAT, both in morphology, as mentioned above, and function. BAT specializes in non-shivering thermogenesis: cold exposure as well as noradrenergic stimulation lead to β-adrenergic excitation, inducing lipolysis with the generation of fatty acids. In BAT, the uncoupling protein 1 (UCP1) represents a way to dissipate the proton gradient in the inner mitochondrial membrane to produce heat during oxidative phosphorylation. WAT also participates in thermogenesis by both providing fatty acids for muscle metabolism (and indirectly heat production) and by directly generating heat after the so called “browning”. The term browning indicates the emergence of beige adipocytes in WAT and it is a process occurring in response to thermogenic need and exercise. Browned (containing beige adipocytes) WAT resembles BAT morphology and metabolism. Similar to BAT, beige fat can produce heat. A fundamental feature of brown/beige adipocytes is the high content in mitochondria, compared with white adipocytes. Mitochondria are essential for cellular energy production but also for differentiation of adipose precursor cells to white and brown adipocytes and the formation of beige cells [33]. NAD(H) and their phosphorylated forms, NADP(H), are crucial coenzymes in mitochondrial oxidative metabolism but also in lipid metabolism.

Recruitment and activation of the beige fat type have the potential to increase energy expenditure, countering obesity and its metabolic complications. Indeed, increasing BAT activity and the conversion of white adipocytes to brown fat-like cells (browning) have been found to protect from diet-induced obesity and insulin resistance in many rodent models [33,34]. A number of studies suggest that browning might represent a promising strategy to alleviate metabolic disturbances also in humans [33]: (i) BAT can act as an anti-diabetic organ by improving insulin sensitivity [35]; (ii) beige adipocytes in WAT are essential to maintain whole-body metabolic homeostasis during catabolic conditions [36,37]; (iii) BAT activity has a significant negative correlation with BMI [38] and; (iv) subjects with an active BAT exhibit improved metabolic health compared with people with reduced BAT [39,40]. Thus, the loss of BAT and beige cell function can contribute to the development of insulin resistance and hyperlipidemia. In humans, the browning process is stimulated by different factors, including sympathetic stimulation upon cold exposure, beta adrenergic receptor activators and the PPAR agonist thiazolidinedione. However, these browning-inducing methods are not appropriate as therapeutic strategies: the effect of cold exposure is reversible and difficult to implement, and the other agonists/compounds induce adverse side effects. Some dietary natural compounds, such as abscisic acid, were suggested as useful aid to induce the browning process [41]. Overall, mechanisms to induce long-lasting WAT browning are highly demanded in humans [33,39,42], because increased brown adipogenesis and WAT browning are considered promising new means for increasing energy expenditure, and are suggested as potential therapeutic strategies to combat obesity and related metabolic disorders, including type 2 diabetes (T2D). Indeed, agents capable of inducing WAT browning recently attracted the interest of researchers and stakeholders in the biomedical, nutritional and pharmaceutical fields.

## 2. The Role of CD38 in Adipose Tissue during Pathophysiological Conditions

### 2.1. CD38 and Obesity

As mentioned above, blocking NAD^+^ consumption by CD38 inhibition represents a strategy to boost NAD^+^ levels. Several studies reported that CD38 knockout mice are protected against high-fat diet-induced obesity, hyperglycemia, and hyperinsulinemia as a consequence of enhanced energy expenditure [43]. The pharmacological inhibition of CD38 recreates the effects observed by genetic deletion, and improves several physiological and metabolic parameters. Specifically, administration of apigenin, a CD38 inhibitor, to obese mice increases NAD^+^ levels and improves glucose and lipid homeostasis [23] and a new potent CD38 inhibitor, 78c, improves several physiological and metabolic parameters, including glucose tolerance [44]. The beneficial outcomes of CD38 absence or inhibition seem to be a consequence of enhanced energy expenditure, and this effect is mediated at least in part via a NAD^+^-dependent activation of SIRT-PGC1α axis, involved in the regulation of mitochondrial biogenesis and energy homeostasis [43].

### 2.2. CD38 and Inflammation in Adipose Tissue during Aging

Chronic, sterile, low-grade inflammation, called inflammaging, develops during aging and is one of the key drivers in the pathogenesis of age-related diseases [45]. Inflammaging negatively influences important metabolic processes such as glucose and lipid homeostasis and insulin sensitivity through a dysregulated communication between innate and adaptive immune cells and metabolic cells such as adipocytes and hepatocytes [46]. Recent studies suggest that inflammaging can also drive the decline in NAD^+^ levels during ageing by a senescence-induced infiltration of CD38^+^ inflammatory cells in metabolic tissues such as WAT and liver [47,48].

Covarrubias et al. showed that during ageing, an accumulation of pro-inflammatory M1-like resident macrophages in the visceral fat and liver is associated with the decline of tissue NAD^+^ levels. In their study, they demonstrated that pro-inflammatory (M1) macrophage polarization is characterized by an increased expression of CD38 and enhanced NAD^+^ consumption whereas anti-inflammatory (M2) macrophage polarization is associated with an increase in NAD^+^ levels. Through in vitro experiments, they showed that by blocking nicotinamide phosphoribosyltransferase (NAMPT) activity, the key enzyme in the salvage pathway, both M1 macrophages and M2 macrophages have a significantly reduced expression of genes associated with their respective phenotypes and this effect can be counteracted by the supplementation of NAD^+^ precursors, NMN and NR. Moreover, they provided evidence that pro-inflammatory cytokines, in particular TNF-α, IL-6, and IL-10, secreted by senescent cells in WAT and liver as a consequence of the senescence-associated secretory phenotype (SASP), are necessary and sufficient to increase the expression of CD38 in macrophages and promote their proliferation [47].

Importantly, Chini et al. (2020), also independently showed that CD38^+^ inflammatory cells accumulate in vivo during aging and this increase is mediated, in part, by the inflammaging induced by senescent cells and their SASP that induce CD38 expression in immune cells and promote the CD38-dependent NAD^+^ decline in tissues. In their work, they demonstrated that multiple subsets of CD38^+^ inflammatory cells (T cells and macrophages) accumulate during aging in WAT and liver and are observed in immune clusters. Ablation of senescent cells or SASP in vivo decreased expression of CD38 and reversed tissue NAD^+^ decline in WAT. Lastly, with in vitro experiments, they proved that blocking the ecto-enzymatic activity of CD38 can increase NAD^+^ through a nicotinamide mononucleotide (NMN)-dependent process. Indeed, the co-treatment with ab68, an anti-CD38 antibody, augmented the NAD^+^-boosting effects of NMN in WAT of both young and old mice.

In summary, the results from both articles provide insight to link the accumulation of senescent cells with age to the age-related NAD^+^ decline through a CD38-mediated mechanism in WAT.

### 2.3. CD38 and Adipose Tissue during Thermogenesis

**Figure 1:**
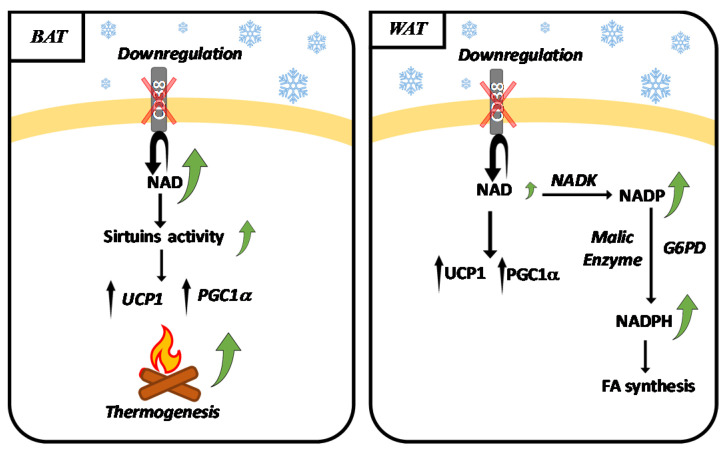
Cold exposure induces the downregulation of CD38 expression in BAT and WAT. During thermogenesis, CD38 expression is downregulated, both in BAT and WAT and this is paralleled by an increase in NAD^+^ levels in BAT, supporting sirtuins’ activity. In WAT, NAD^+^ is converted to NADP^+^ and NADPH as a consequence of increased expression of NAD^+^ kinase (NADK), G6PD (Glucose-6-phosphate dehydrogenase), and malic enzyme. NADPH in WAT sustains FA (fatty acid) synthesis.

Recently, Benzi et al. conducted a study to evaluate the role of CD38 in thermogenesis [49], a fundamental aspect of energy homeostasis. A scheme showing the effect of CD38 downregulating in BAT and WAT during thermogenesis is shown in Figure 1.

The gene expression levels of the master regulator of mitochondrial activity Pgc-1α, and of the pivotal browning marker Ucp1 were significantly higher in BAT and WAT from Cd38^-/-^ mice kept at 6 °C, compared with wild-type mice. Moreover, the hormone-sensitive lipase (HSL) activation was also enhanced in BAT from cold-exposed Cd38^-/-^ mice, compared with the controls. Thus, the absence of CD38 seems to potentiate the browning process. The absence of CD38 in adipose tissue was paralleled by a high content of NAD^+^, confirming that CD38 is a major consumer of NAD^+^ in both brown and white adipose tissue: the absence of CD38 increased NAD^+^ content by more than two-fold in BAT, and by six-fold in WAT [49]. Interestingly, the cold-exposure increased NAD^+^ content in BAT from wild-type mice, indicating that the NAD^+^-related metabolism is important in thermogenesis and possibly reflects the increased demand for NAD^+^ in mitochondria-rich BAT. Recently, a role for glucose oxidation in brown fat thermogenesis was demonstrated [50]. Increased levels of NAD^+^ may also be useful for glucose oxidation and flux into the mitochondrial TCA cycle.

A similar result in terms of NAD^+^ increase in BAT upon cold exposure was obtained also in a study conducted by Dr Yoshino’s group [51]. The NAD^+^ increase was reported to occur as a consequence of an enhanced Nampt gene expression [51]. Although Nampt overexpression in BAT from cold-exposed mice was also confirmed in our study, we did not observe an increased NAD^+^ synthesis in BAT during thermogenesis. Instead, we found that cold exposure caused a significant decrease in the expression level of CD38. The reduction in CD38 expression was confirmed at the mRNA and protein level, as well as by measuring CD38 enzymatic activities (namely, NAD^+^-ase, ADP-ribosyl cyclase, and GDP-ribosyl cyclase) [49]. CD38 markedly affects NAD^+^ content, and the CD38 expression was demonstrated to inversely correlate with the intracellular NAD^+^ content in many cell systems/organs, including cell lines [52], and lung, kidney, brain [53], and WAT and BAT from Cd38^-/-^ mice [49]. By cell dissociation and FACS analysis, we also demonstrated that the downregulation of CD38 is occurring in brown adipocytes, and not in other cell types found in the tissue. Nevertheless, the percentage of CD45^+^ leukocytes infiltrating the BAT was reduced upon cold exposure, but the frequency of CD38^+^ cells was not reduced in the CD45^+^ population. Altogether, our data indicate that the decrease in CD38 expression in BAT during cold exposure is due to a downregulation in CD38 expression in adipocytes, and to a reduction in the number of CD45^+^/CD38^+^ infiltrating cells. The latter observation may suggest that in obesity, a condition often associated with WAT chronic inflammation, which determines insulin resistance, and the presence of inflammatory CD38^+^ cells [54] might interfere with the browning process.

Downregulation of CD38 at the mRNA, protein and enzymatic levels also occurred in WAT from cold exposed mice [49]. In WAT, the CD38 decrease was paralleled by a small increase in NAD^+^ whereas a markedly higher increase in NADP^+^ and NADPH was detected, suggesting that NAD^+^ is re-directed towards its phosphorylated oxidized and reduced forms during cold-induced adaptation [49]. In line with this hypothesis, the expression of NAD kinase (synthesizing NADP^+^ from NAD^+^), G6PD and malic enzyme (both enzymes reducing NADP^+^ to NADPH) were found to be increased in WAT [49].

In BAT, the increased levels of NAD^+^ likely sustain the activity of NAD^+^-dependent deac(et)ylases (sirtuins). Indeed, SIRT1, SIRT3, and SIRT6 were demonstrated to regulate BAT activity. More specifically, in line with the role of SIRT1 in energy expenditure, overexpression of SIRT1 enhances insulin sensitivity by potentiating BAT function and energy expenditure, and this effect was demonstrated to derive from a higher response to β3-adrenergic stimuli [55]. Moreover, SIRT1 deficiency induces whitening of BAT by reducing thermogenic genes and ETC complexes [55,56]. Mitochondrial SIRT3 regulates UCP1 and PGC1α expression and oxygen consumption in brown adipocytes [57]. However, the role of SIRT3 in thermogenesis is controversial: on one hand, whole-body Sirt3-deficient mice and adipocyte-specific mitochondrial Sirt3 knockout mice were reported to have normal BAT function and cold tolerance [58]; conversely, Sebaa et al. demonstrated that mice deficient in SIRT3 display widespread defects in BAT lipid use/oxidation and in thermoregulation, with SIRT3 indirectly controlling BAT thermogenesis by affecting pathways upstream of UCP1 [59]. Finally, SIRT6 expression is induced by cold exposure and β-adrenergic stimulation. Deletion of SIRT6 in adipose tissue impairs the thermogenic function of BAT, causing BAT “whitening” [60].

The fact that CD38 activity is decreased, suggests that the CD38-produced Ca^2+^ mobilizing second messengers, cADPR and ADPR (from NAD^+^), may not be required during cold-induced thermogenesis. Possibly in line with this view, increased [Ca^2+^]_i_ levels are expected to decrease hormone-sensitive lipase (HSL) phosphorylation, and thereby lipolysis [61]. However, the detailed role for NAD^+^-dependent regulation of Ca^2+^ homeostasis in browning must be investigated. In fact, the decrease in CD38 expression may be paralleled by an increase in the levels of the Ca^2+^ mobilizing second messenger NAADP^+^, which was reported to be degraded by CD38 [7].

To the best of our knowledge, cold-induced thermogenesis in BAT and WAT is the first “physiological” process accompanied by a decrease in CD38 expression. Conversely, CD38 upregulation was reported to occur in various physiological processes, likely to regulate cADPR/ADPR production and Ca^2+^ homeostasis [13,62,63,64,65]. Different cytokines upregulate CD38 expression in airway smooth muscles, and this process is involved in asthma pathogenesis [13]. The microRNAs, miR-140-3p and miR-708, down-regulate CD38 overexpression induced by TNFα in airway smooth muscle cells [66,67]. In the study by Benzi et al., the authors unveiled that miR-140-3p expression is increased in BAT during thermogenesis, suggesting that this may be a possible mechanism causing CD38 downregulation [49]. However, a causal link between miR-140-3p and CD38 expression must be further investigated in adipose tissue. Different miRNAs regulate the browning process, and may be relevant in the modulation of CD38 expression as well [68]. The possible involvement of cold shock proteins in the regulation of CD38 expression during thermogenesis should be investigated. Cold shock proteins are multifunctional RNA/DNA binding proteins, which regulate transcription, splicing, translation, and the exosomal RNA content [69].

### 2.4. NADK, G6PD, Malic Enzyme and Browning

As mentioned above, along with the downregulation of CD38 expression, cold exposure upregulates the expression of the cytosolic form of NADK in WAT, determining an increased conversion of NAD^+^ to NADP^+^. In the winter rape plant, cold exposure was reported to upregulate NADK activity [70]. In mammals, very few studies demonstrated the regulation of NADK expression and/or activity. In WAT, the mRNA levels of the mitochondrial Nadk (mNadk) are regulated by nutrition levels [71]. Cold exposure might metabolically “resemble” fasting in WAT, and fasting increased mNadk levels [71]. The possible regulation of mNadk in WAT during browning needs to be investigated.

In WAT, Benzi et al. reported, for the first time, that G6PD and malic enzyme expression were also upregulated upon cold exposure, with a consequent increase in NADPH level. We speculated that increased levels of NADPH are needed during thermogenesis to sustain fatty acid synthesis [72,73], as the enzymes necessary to this process, acetyl-CoA carboxylase and fatty acid synthase, were both found to be upregulated in WAT from mice exposed to cold temperatures. The detailed mechanisms regulating Nadk, G6pdx, and malic enzyme expression, and thus the destiny of the NAD(P) pool, are to be defined.

The effect on the NADP(H) level of CD38 pharmacological inhibition was investigated in an ex vivo model of myocardial ischemia/reperfusion injury: endothelial NADP(H) levels were greatly decreased in postischemic conditions, whereas in the presence of 78c (a CD38 inhibitor), the decrease in NADP(H) levels, was greatly reduced [74].

Notably, the cold-induced increase in NADPH is observed in WAT but not in BAT, indicating the higher demand for NADPH for anabolic processes necessary for the vascularization and differentiation of adipocyte precursors observed during cold exposure in WAT [75].

## 3. Conclusions and Perspective

**Figure 2:**
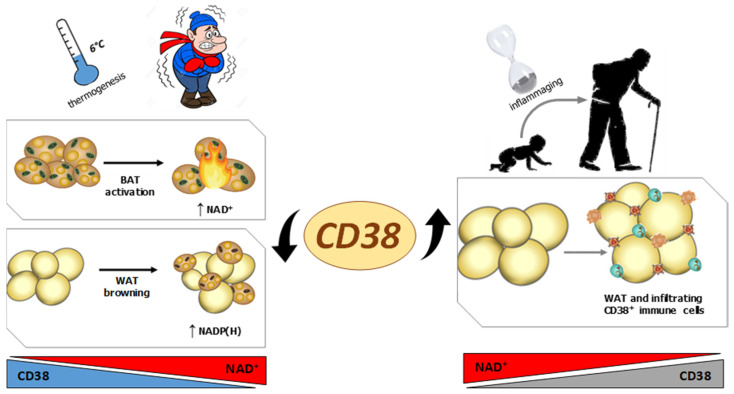
Regulation of CD38 expression and NAD(P) content in adipose tissue during thermogenesis or during inflammaging. CD38 expression is regulated in opposite directions during thermogenesis or during inflammaging. In thermogenesis, CD38 downregulation is paralleled by an increase in NAD^+^ levels in BAT, and by an increase in NADPH in WAT. During inflammaging, CD38 upregulation is followed by a NAD^+^ decrease in WAT.

In the study by Benzi et al. [49], NAD^+^ increase in BAT during cold-induced thermogenesis was ascribed to CD38 downregulation (Figure 1 and Figure 2). Instead, Yoshino et al., related the increased levels of NAD^+^ in BAT to Nampt overexpression, by employing the use of brown adipocyte-specific Nampt knockout mice to explore the key role of NAD^+^ synthesis [51]. Overall, the regulation of NAD^+^ synthesis, availability, and metabolism is a key event during thermogenesis in BAT and WAT. An NAD^+^ deficiency in WAT, due to an increased presence of CD38^+^ inflammatory cells, may impair crucial metabolic functions, particularly in obese and/or older adults (Figure 2).

An increased NAD^+^ availability to enhance adipose tissue function and energy metabolism may be achieved by the administration of NAD^+^ precursors, such as NMN, NR, or their reduced forms. Moreover, pharmacological inhibition of CD38 was suggested as a possible therapeutic strategy against obesity and type 2 diabetes. Increasing BAT activity and WAT browning is expected to enhance the expenditure of excess stored energy, counteracting obesity and related inflammatory and/or metabolic disorders. In principle, CD38 inhibition may be considered a more efficient strategy to increase BAT activity: indeed, other supplements boosting NAD^+^ also generate increased levels of the CD38 substrate, which is likely not a desired effect.

Elucidating the mechanisms behind BAT activation and the process of “browning” is of great interest for developing therapeutic strategies against obesity.

## Data Availability

Not applicable.
